# Development of a diet-induced murine model of diabetes featuring cardinal metabolic and pathophysiological abnormalities of type 2 diabetes

**DOI:** 10.1242/bio.016790

**Published:** 2016-07-11

**Authors:** Jodie L. Morris, Tahnee L. Bridson, Md Abdul Alim, Catherine M. Rush, Donna M. Rudd, Brenda L. Govan, Natkunam Ketheesan

**Affiliations:** Australian Institute of Tropical Health and Medicine, Division of Tropical Health and Medicine, James Cook University, Townsville, Queensland 4811, Australia

**Keywords:** Type 2 diabetes, Murine model, Diet-induced, Glucose intolerance, Insulin resistance

## Abstract

The persistent rise in global incidence of type 2 diabetes (T2D) continues to have significant public health and economic implications. The availability of relevant animal models of T2D is critical to elucidating the complexity of the pathogenic mechanisms underlying this disease and the implications this has on susceptibility to T2D complications. Whilst many high-fat diet-induced rodent models of obesity and diabetes exist, growing appreciation of the contribution of high glycaemic index diets on the development of hyperglycaemia and insulin resistance highlight the requirement for animal models that more closely represent global dietary patterns reflective of modern society. To that end, we sought to develop and validate a murine model of T2D based on consumption of an energy-dense diet containing moderate levels of fat and a high glycaemic index to better reflect the aetiopathogenesis of T2D. Male C57BL/6 mice were fed an energy-dense (ED) diet and the development of pathological features used in the clinical diagnosis of T2D was assessed over a 30-week period. Compared with control mice, 87% of mice fed an ED diet developed pathognomonic signs of T2D including glucose intolerance, hyperglycaemia, glycosylated haemoglobin (HbA_1c_) and glycosuria within 30 weeks. Furthermore, dyslipidaemia, chronic inflammation, alterations in circulating leucocytes and renal impairment were also evident in ED diet-fed mice compared with mice receiving standard rodent chow. Longitudinal profiling of metabolic and biochemical parameters provide support of an aetiologically and clinically relevant model of T2D that will serve as a valuable tool for mechanistic and therapeutic studies investigating the pathogenic complications of T2D.

## Abstract (precis)

**Summary:** We describe a murine model of type 2 diabetes based on chronic consumption of an energy-dense diet. Longitudinal sampling demonstrates metabolic and pathological changes closely simulating progression to overt diabetes.

## INTRODUCTION

Type 2 diabetes (T2D) continues to be a global health priority with a significant proportion of health care budgets directed to management of T2D and its complications. Between 2010 and 2030, the percentage of adults with T2D is expected to increase by 69% in developing countries and 20% in developed countries (Shaw et al., 2010). T2D is a multifarious, progressive disease involving a dynamic interplay of lifestyle factors and genetic predisposition that contribute to changes in glucose metabolism, with overt T2D typically taking years to develop. The transition from pre-diabetic states to T2D involves the progressive failure of pancreatic beta (β)-cells, involving reduced β-cell mass and increased apoptosis. Persistently elevated blood glucose levels enhance pancreatic β-cell dysfunction resulting in a more rapid functional deterioration and a non-compensatory, hyperglycaemic state which is a key diagnostic feature of T2D (Fonseca, 2009). Vascular complications associated with T2D arise from a combination of prolonged hyperglycaemia, advanced glycation end products (AGE) and oxidative stress, culminating in chronic inflammation and altered cellular function within many organ systems (Forbes and Cooper, 2013). Patients with T2D are at increased risk of developing nephropathy, retinopathy, neuropathy and cardiovascular diseases including atherosclerosis, myocardial infarction and cerebral ischaemic accidents (Forbes and Cooper, 2013). Moreover, there is growing appreciation of the convergence of T2D and communicable diseases such as tuberculosis, which also account for a significant proportion of global morbidity and mortality (Bridson et al., 2014; Hodgson et al., 2015).

Rodent models have contributed significantly to our understanding of insulin resistance, obesity and diabetes (King and Bowe, 2015). Genetically altered murine models, such as *Lept^db^* mice, fail to incorporate the significant nutritional and polygenic determinants involved in the complex pathogenesis of clinical T2D. In contrast, diet-induced rodent models of T2D are considered more akin to the clinical aetiopathology of this disease. However, there is substantial discordance in the metabolic phenotypes reported between studies using diet-induced models, which is confounded by differences in dietary composition and fat content, age of mice and duration of feeding, along with the rodent strain and gender (King and Bowe, 2015). Several studies have also combined a diet-induced rodent model with streptozotocin-induced destruction of pancreatic β-cells to shorten the length of time required for development of signs of overt diabetes (Gilbert et al., 2011; King and Bowe, 2015). The caveat of such models however, is that the micro- and macro-vascular complications associated with clinical T2D require significant time to become established and cannot be achieved in a short-term treatment regime. In addition, such models have limited utility due to inadvertent toxicity of streptozotocin on renal and hepatic tissue (Deeds et al., 2011).

The high-fat diet (HFD)-fed C57BL/6 model introduced by Surwit et al. (1988) is used widely as a model of obese T2D; however, a criticism of this diet is that the fat content (60% of energy) markedly exceeds typical dietary intake in developed nations (34% of energy) (Harika et al., 2013). Over the past decade it has become increasingly apparent that consumption of refined carbohydrates is also an important contributor for the development of metabolic dysfunction. Energy-dense (ED) diets, characterised by higher intake of processed and red meat, high-fat foods, sugary desserts and drinks positively correlate with the incidence of cardiovascular disease, cancer and T2D (Malik and Hu, 2015; Riccardi et al., 2008; Rizkalla, 2014). The high glycaemic index associated with ED diets has the potential to predispose individuals to hyperinsulinaemia, which is thought to result in pancreatic β-cell exhaustion and subsequent relative insulin deficiency (Andersson et al., 2010; Pawlak et al., 2004). Recently, we investigated the impact of an ED diet (23% and 50.5% total energy from fat and refined carbohydrates, respectively) on the development of hyperglycaemia and glucose intolerance in C57BL/6 mice (Hodgson et al., 2013). Progression to hyperglycaemia and glucose intolerance occurred more rapidly for mice consuming the ED diet compared with those fed standard (SD) chow, suggesting that this may be a useful and relevant method for developing a model of T2D (Hodgson et al., 2013). Therefore, in the current study we evaluated whether ED feeding over a period of 30 weeks emulates the development of key metabolic and pathophysiological features associated with progression to overt T2D. Our findings demonstrate that C57BL/6 mice fed an ED diet for up to 30 weeks develop metabolic and immunological dysfunction that closely simulates the natural progression of T2D.

## RESULTS

### ED diet-fed mice develop increased body and adipose tissue mass

**Fig. 1.**
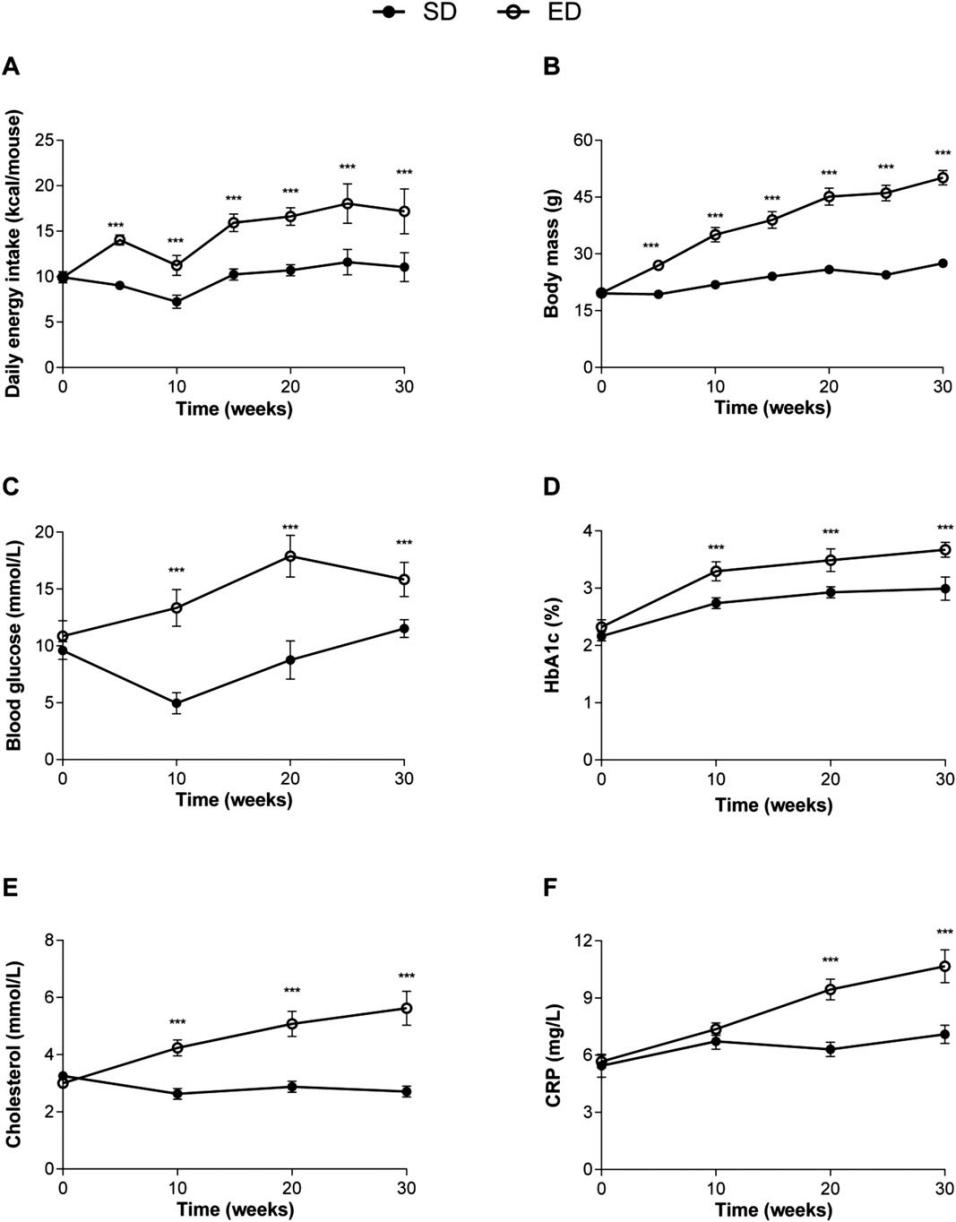
**Increased caloric intake corresponds with progressive weight gain, hyperglycaemia, formation of advanced glycation end products, dyslipidemia and serum inflammatory markers in mice fed an energy-dense (ED) diet for 30 weeks.** (A) Daily energy intake and (B) weight gain of mice fed a standard (SD; *n*=22) and an ED (*n*=23) diet for 30 weeks. Compared with controls, fasting plasma (C) glucose concentrations and (D) glycosylated haemoglobin (HbA_1c_) were significantly higher in ED diet-fed mice throughout the study period. Mice consuming the ED diet also had significantly increased (E) plasma cholesterol and (F) C-reactive protein (CRP) at 10, 20 and 30 weeks when compared with control animals. Data are mean±95% c.i. Significant differences were analysed by two-way ANOVA and Sidak's multiple comparison post tests and indicated as ***P≤0.001.

Prior to diet intervention, body mass was comparable between groups (SD, 19.7±0.4 g vs ED, 19.6±0.4 g; mean±95% confidence interval). Daily energy intake was significantly higher (1.5-fold) for animals fed an ED diet (14.7±3.1) compared with SD diet controls (9.9±1.5, Fig. 1A) and corresponded with significantly increased total body mass for ED diet-fed mice compared with controls (Fig. 1B) over the 30 week intervention. An overall increase of 28.85% and 60.75% total body weight was observed for mice fed SD or ED diet, respectively, for 30 weeks (Fig. 1B). After 30 weeks of feeding, ED diet mice also demonstrated greater visceral (∼sixfold, epididymal and retroperitoneal) and subcutaneous (∼ninefold, inguinal) fat pad mass when compared with control mice fed SD diet (Table 1).Wet tissue weights of liver, spleen and pancreas were significantly greater in mice fed an ED diet for 30 weeks compared with control mice that received a SD diet (Table 1). There was no significant difference in wet lung mass for either dietary group after 30 weeks of feeding.

**Table 1.**
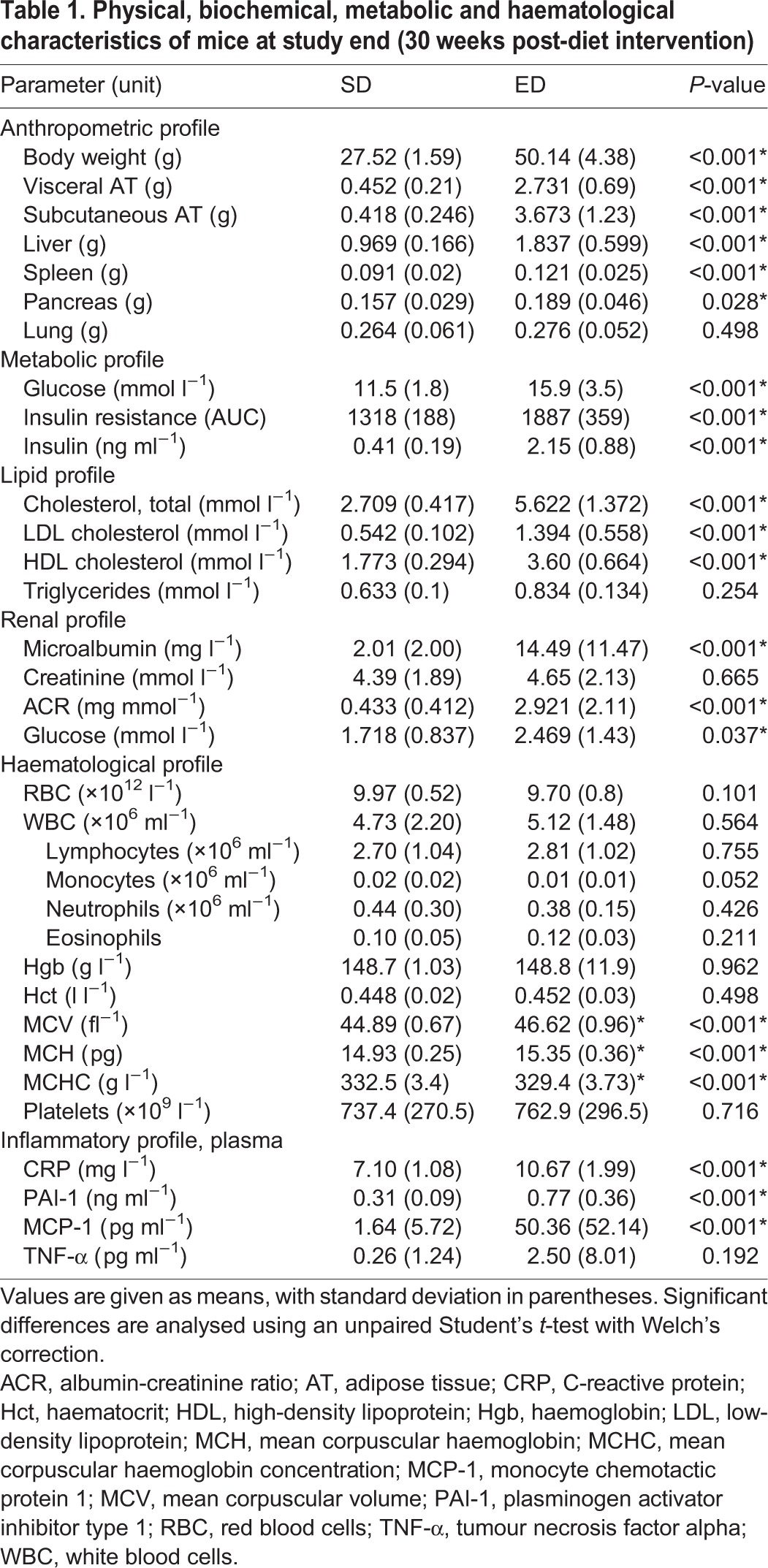
**Physical, biochemical, metabolic and haematological characteristics of mice at study end (30 weeks post-diet intervention)**

### ED diet feeding results in increased fasting blood glucose, glycosylated haemoglobin (HbA_1c_) and glucose intolerance

**Fig. 2.**
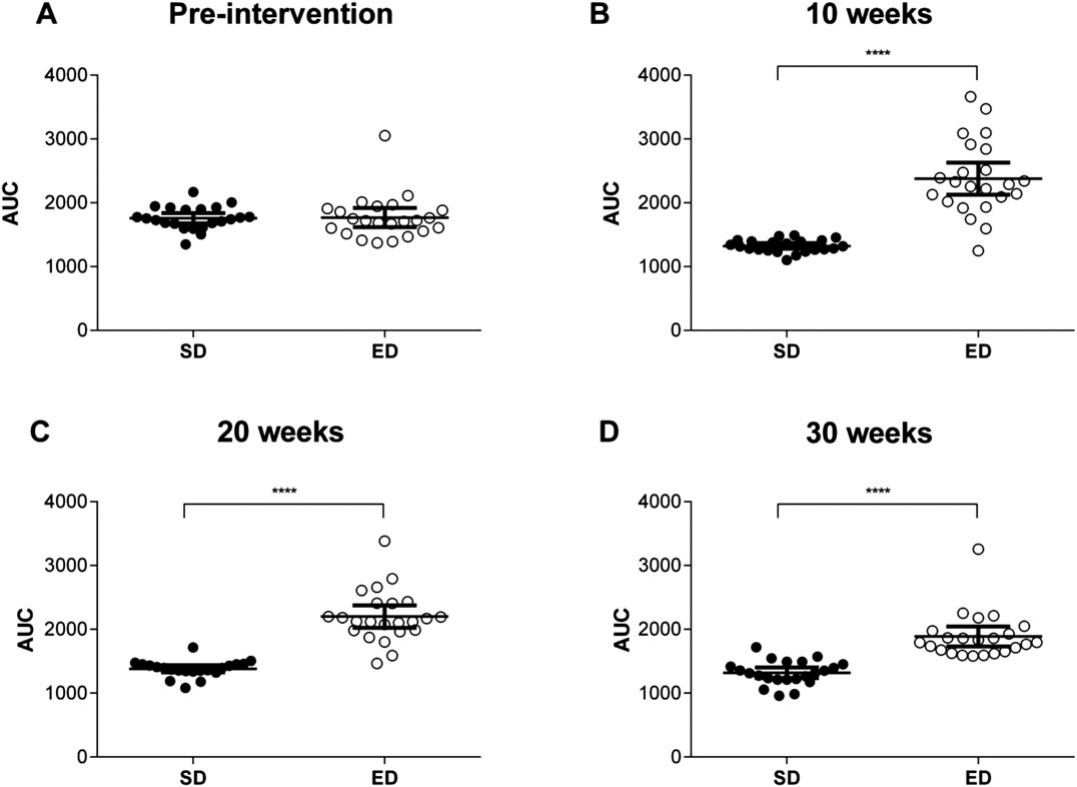
**Impaired glucose tolerance in mice fed an energy-dense (ED) diet.** At baseline (A), 10 weeks (B) 20 weeks (C) and 30 weeks (D) after commencement of dietary intervention, glucose concentrations were measured at 15, 30, 60 and 120 min post intraperitoneal glucose load (2 g kg^−1^) for control mice fed a standard (SD; *n*=22) and ED (*n*=23) diet and the area under the curve (AUC) for glucose levels following the glucose tolerance test (GTT) determined. Data are mean±95% c.i. Significant differences were analysed by two-way ANOVA and Sidak's multiple comparison post-tests for GTT and Student's *t*-test for AUC and indicated as ***P≤0.001.

Prior to dietary intervention, fasting blood glucose levels were comparable between groups (SD, 9.6±1.7 mmol l^−1^ and ED, 10.8±3.1 mmol l^−1^; *P*=0.10). However, compared with control mice, fasting blood glucose levels were elevated in ED diet mice by 10 weeks after commencement on the diet and remained significantly higher throughout the intervention period (Fig. 1C). Despite comparable levels at baseline, glycated haemoglobin (HbA_1c_) concentrations were significantly increased in ED diet-fed mice by 10 weeks (3.3±0.4 vs 2.7±0.2) and remained higher than SD diet controls throughout the intervention (Fig. 1D). ED diet-fed mice had significantly impaired glucose tolerance when compared with control mice (Fig. 2). The area under the curve (AUC) derived from the intraperitoneal glucose tolerance test (GTT) was higher for ED diet- than SD diet-fed mice at 10, 20 and 30 weeks of the intervention. These data demonstrate significant metabolic changes within 10 weeks of ED diet feeding with progressive deterioration in the ability to maintain normoglycaemia and steady increases in HbA_1c_, a key diagnostic marker for glycaemic control (American Diabetes Association, 2014; The Royal Australian College of General Practitioners and Diabetes Australia, 26 Nov 2015, http://www.racgp.org.au/your-practice/guidelines/diabetes/3-screening,-risk-assessment,-case-finding-and-diagnosis/34-diagnosis-of-diabetes), across the study period.

### ED diet-fed mice develop hyperinsulinaemia and insulin resistance

**Fig. 3.**
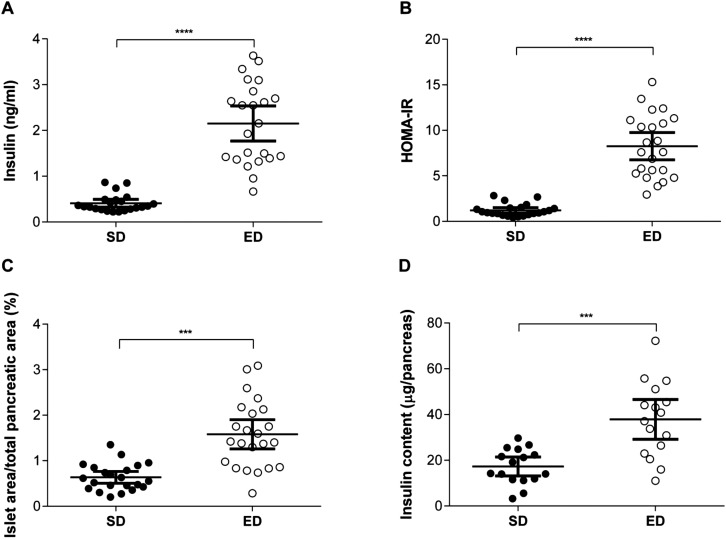
**Hyperinsulinemia and pancreatic changes in energy dense (ED) diet-fed mice.** (A) Fasting plasma insulin concentrations (ng ml^−1^) were significantly higher in mice fed an ED (*n*=23) diet for 30 weeks, compared with control mice fed a standard (SD; *n*=22) diet. (B) Moreover, HOMA-IR values, as an indicator of insulin resistance, were greater in ED diet-fed mice in comparison with SD control mice. (C) Pancreatic islet area was measured and expressed as a percentage of the total pancreatic area per section for each mouse. Compared with control mice, hyperplasia of islet area was evident in pancreas from mice fed an ED diet for 30 weeks. (D) Total insulin levels in pancreas homogenates from mice fed an ED diet for 30 weeks were elevated compared with levels in control mice. Data are mean±95% c.i. Significant differences were analysed by Student's *t*-test with Welch's correction and indicated as ***P≤0.001.

At the end of the study period (30 weeks), fasting plasma insulin levels were fivefold higher in ED diet-fed mice compared with controls (Fig. 3A). Furthermore, insulin secretion in response to glucose challenge was significantly increased and persistently higher for mice fed an ED diet compared with SD diet controls (Fig. S1). The homeostasis model assessment for insulin resistance (HOMA-IR) was used to determine insulin resistance based on the fasted insulin and glucose levels after 30 weeks on the diet. ED diet-fed mice had a 6.8-fold higher HOMA-IR value compared with control mice (Fig. 3B). Consistent with a hyperinsulinaemic state, significant differences were also observed in the islet area within pancreas from SD and ED diet-fed mice (Fig. 3C-E). Compared with control mice, the percentage of pancreatic islet area was increased in mice fed an ED diet. Moreover, total insulin content recovered from pancreas homogenates of ED diet-fed mice was significantly greater than that of mice fed a SD diet (Fig. 3F). Combined, these data demonstrate hyperinsulinemia and insulin resistance in mice fed an ED diet for 30 weeks, together with pathological changes in the pancreas which are consistent with compensatory islet hyperplasia described in patients with T2D (Quan et al., 2013) and other animal models of diabetes (Bock et al., 2003; Guo et al., 2015).

### Haematological and systemic inflammatory changes are induced in response to ED diet consumption

Haematological data for mice fed a SD or ED diet for 30 weeks was compared (Table 1). No significant differences were observed in red blood cell (RBC), white blood cell (WBC) and platelet counts between mice fed SD or ED diet for 30 weeks using a Coulter^®^ Ac-T diff haematology analyser. Similarly, haematocrit and total haemoglobin concentrations were comparable between mice in each dietary group. Compared with control mice, mean corpuscular volume (MCV) and mean corpuscular haemoglobin (MCH) were significantly greater in peripheral blood from ED diet-fed mice. In contrast, mean corpuscular haemoglobin concentration (MCHC) was lower in mice fed an ED diet for 30 weeks compared with those fed a SD diet (Table 1). These data demonstrate that consumption of an ED diet for 30 weeks contributes to alterations in RBC morphology and is consistent with changes described in patients with T2D (Soma and Pretorius, 2015).

**Fig. 4.**
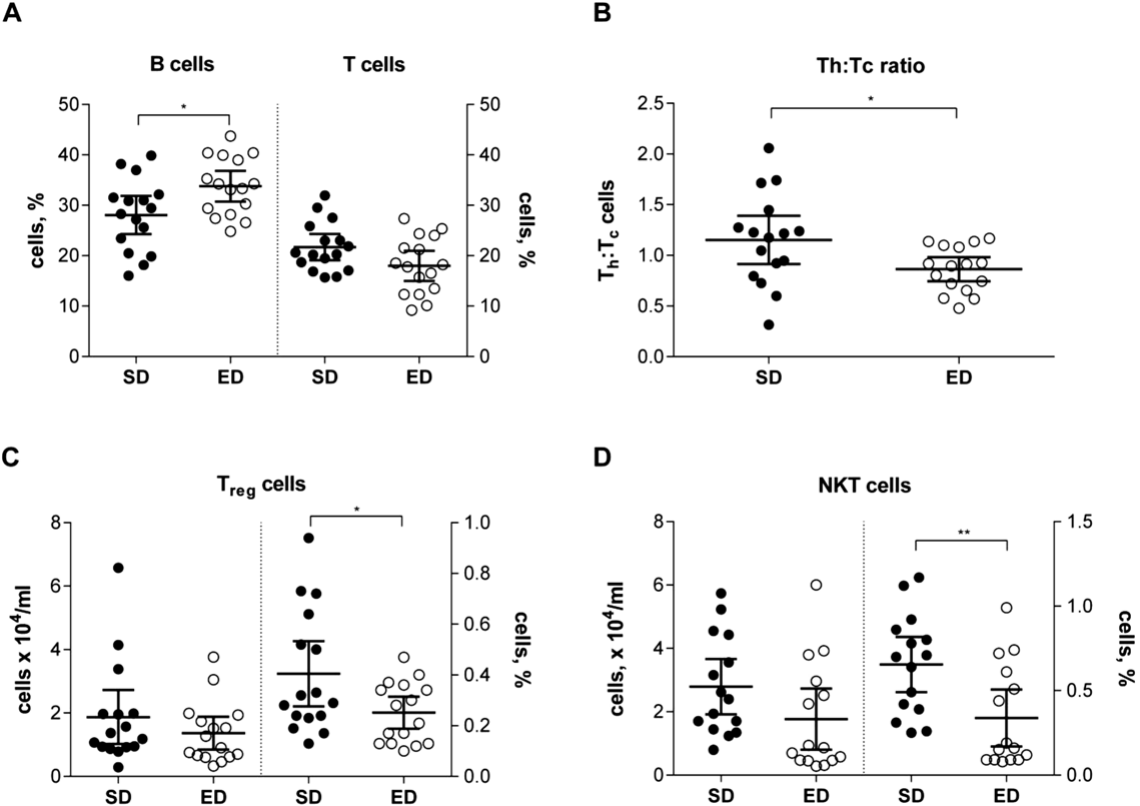
**Altered immune cell profile in blood of mice fed an energy-dense (ED) diet.** Peripheral blood leucocyte frequencies were compared in mice fed a standard (SD; *n*=16) and ED (*n*=15) diet for 30 weeks. (A) Compared with control mice, the percentage of B cells was significantly increased in blood from ED diet-fed mice and corresponded with trend for decreased percentage of total T cells, though this did not reach statistical significance (*P*=0.056). (B) The total number of T_helper_ (T_h_, CD4^+^) cells was reduced in peripheral blood from ED diet-fed mice and was reflected by a significantly lower ratio of T_h_:T_cytotoxic_ (T_c_) cells when compared with mice fed a SD diet for 30 weeks. (C) The proportion of T_regulatory_ cells (T_reg_, CD4^+^CD25^+^) was significantly lower in blood from mice fed an ED diet for 30 weeks, despite comparable absolute numbers of T_reg_ cells between dietary groups (*P*=0.28). (D) Similarly, the percentage of peripheral blood natural killer T (NKT) cells was significantly reduced for ED diet-fed mice with a trend for an overall reduction in the total number of NKT cells (*P*=0.1). Data are mean±95% c.i. Significant differences were analysed by Student's *t*-test with Welch's correction and indicated as *P≤0.05, **P≤0.01.

Given the pivotal role that leucocytes play in the development of T2D and its associated complications including increased susceptibility to infectious diseases (Forbes and Cooper, 2013), we next investigated whether consumption of an ED diet for 30 weeks led to changes in key cells of the innate and adaptive immune response using cell surface marker staining and flow cytometry. Consistent with the findings using the Coulter^®^ haematology analyser, peripheral blood total leucocyte yields were comparable between SD and ED diet-fed mice. No significant differences were observed in either the absolute number or percentage of polymorphonuclear cells (PMN), natural killer (NK) and professional phagocytes in blood from control and ED diet-fed mice (Fig. S2). In contrast, the proportion of B-cells was significantly increased in blood from ED diet-fed mice, corresponding to a trend for a reduction in the percentage of T cells (Fig. 4A). Further elucidation of the frequency of particular T cell subsets demonstrated that the total number of T_helper_ (T_h_, CD4^+^) cells was reduced in peripheral blood from ED diet-fed mice. Consequently, the ratio of T_h_ and T_cytotoxic_ (T_c_, CD8^+^) cells was significantly lower for mice fed an ED diet for 30 weeks, when compared with mice fed a SD diet (Fig. 4B). Moreover, there were also differences in the proportions of T_regulatory_ cells (T_reg_, CD4^+^CD25^+^) and natural killer T-cells (NKT, TCRβ^+^CD1d/α-GalCer dimer^+^), cells in peripheral blood from SD and ED diet-fed mice (Fig. 4C,D). Despite absolute numbers of T_reg_ cells being comparable between dietary groups (*P*=0.28), the percentage of T_reg_ cells was significantly lower in blood from mice fed an ED diet for 30 weeks. Similarly, the percentage of peripheral blood NKT cells was significantly reduced for ED diet-fed mice with a trend for an overall reduction in the total number of NKT cells per ml of blood (*P*=0.1). In summary, these data demonstrate that by 30 weeks, subclinical systemic inflammation is apparent in mice fed an ED diet, consistent with the features of clinical T2D (Donath and Shoelson, 2011; Festa et al., 2006; Khodabandeloo et al., 2015). Furthermore, contrasting leucocyte profiles were demonstrated between SD and ED diet-fed mice, particularly within lymphocyte subsets.

Subclinical chronic inflammation is a hallmark of T2D and is evidenced by elevation in acute phase proteins such as C-reactive protein (CRP) and plasminogen activator inhibitor type 1 (PAI-1) (Festa et al., 2006), and inflammatory cytokines such as tumour necrosis factor alpha (TNF-α), monocyte chemotactic protein 1 (MCP-1) and interleukin (IL)-6 (Kahn et al., 2014; Khodabandeloo et al., 2015). Therefore, using these markers, we compared plasma concentrations in mice fed an ED or SD diet for 30 weeks to assess the development of systemic low-grade inflammation. CRP was significantly higher in response to the ED diet when compared with controls at 10, 20 and 30 weeks of the intervention (Fig. 1F, Table 1). In addition, circulating PAI-1 and MCP-1 levels were significantly elevated in mice fed an ED diet for 30 weeks, compared with SD diet-fed control mice (Table 1). A similar trend was observed for plasma TNF-α levels after 30 weeks of feeding, though this did not reach statistical significance (Table 1). No significant differences were observed in plasma concentrations for IL-6, IL-12p70, interferon gamma (IFN-γ) or IL-10 between SD and ED diet-fed mice at completion of the study period.

### Dyslipidemia and ectopic fat deposition is evident in ED diet-fed mice

Baseline measurements of fasting total (Fig. 1E), low density lipoprotein (LDL)- (Fig. S3A) and high density lipoprotein (HDL)-cholesterol (Fig. S3B) levels in plasma were comparable for both dietary groups. After 10 weeks on the diet, ED diet-fed mice demonstrated significantly elevated total cholesterol levels in comparison with control mice on a SD diet and this trend continued over the remainder of the 30-week intervention (Fig. 1E). Similarly, LDL- and HDL-cholesterol levels were 1.2- and 1.7-fold higher, respectively in ED diet-fed mice by 10 weeks after diet intervention with these differences persisting to 30 weeks (Fig. S3A,B). At 10 and 20 weeks post-dietary intervention, circulating triglycerides were significantly elevated in plasma from ED diet-fed mice compared with mice fed SD diet (Fig. S3C). However, by 30 weeks plasma triglyceride levels were comparable for SD and ED diet-fed mice (Table 1, Fig. S3C).

**Fig. 5.**
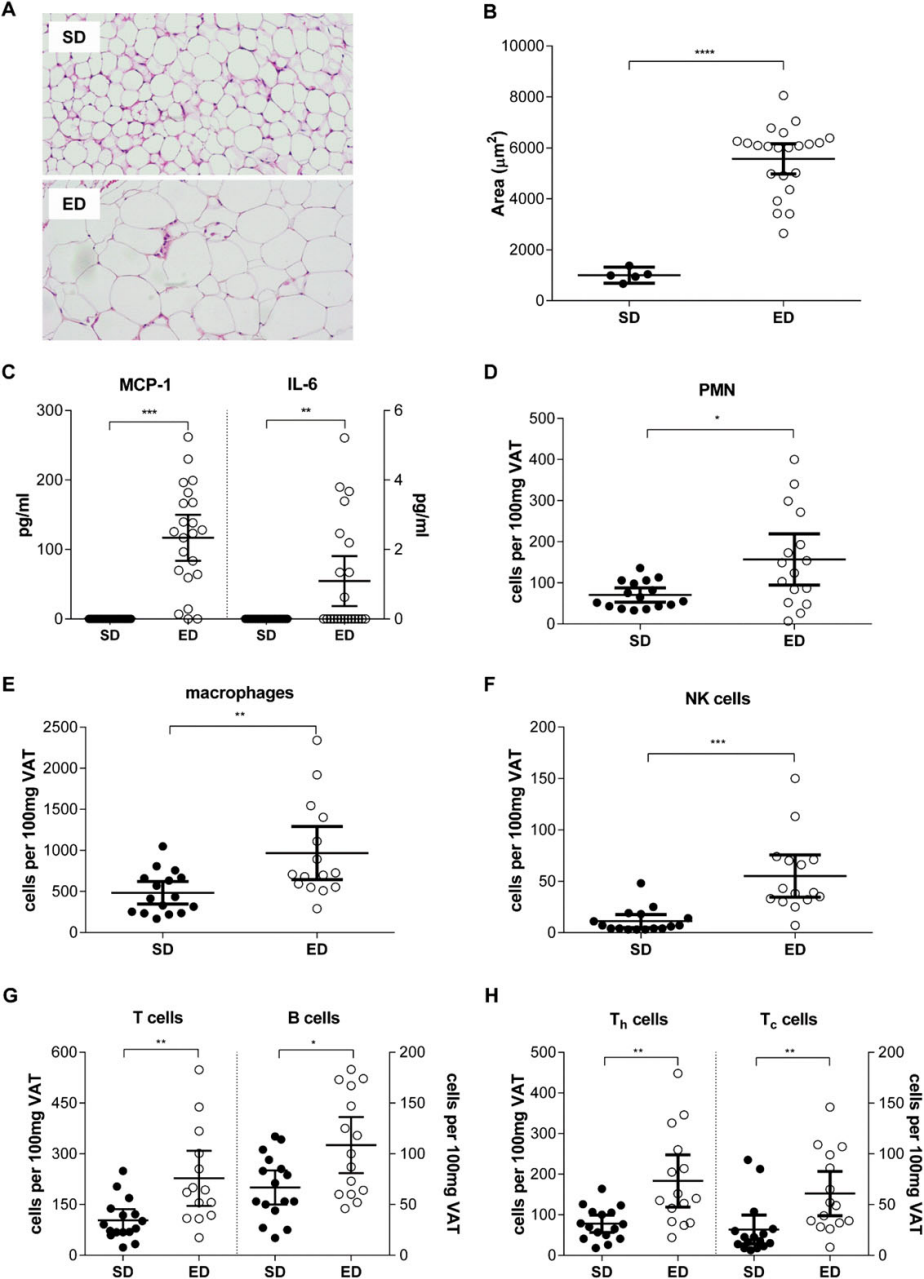
**Visceral adipose tissue pathology in energy dense (ED) diet-fed mice.** (A) Representative photomicrographs of visceral adipose tissue (VAT) from standard (SD; *n*=22) and ED (*n*=23) diet-fed mice after 30 weeks. (B) Mean adipocyte size (μm^2^) was significantly greater in VAT from mice fed an ED diet, compared with those fed a SD diet. (C) Concentrations (pg ml^−1^) of the inflammatory cytokines monocyte chemotactic protein 1 (MCP-1) and interleukin (IL)-6 were markedly higher in VAT of mice fed an ED diet for 30 weeks, compared with SD diet-fed mice. Consistent with this, comparison of equivalent masses (100 mg) of VAT from SD (*n*=16) and an ED (*n*=15) diet-fed mice demonstrated significant differences in the frequencies of (D) PMN, (E) macrophages, (F) NK cells, (G) B and T cells and (H) T_helper_ (T_h_) and T_cytotoxic_ (T_c_) cells within the stromal vascular fraction. Data are mean±95% c.i. Significant differences were analysed by Student's *t*-test with Welch's correction and indicated as **P≤0.01, ***P≤0.001, ****P≤0.0001.

**Fig. 6.**
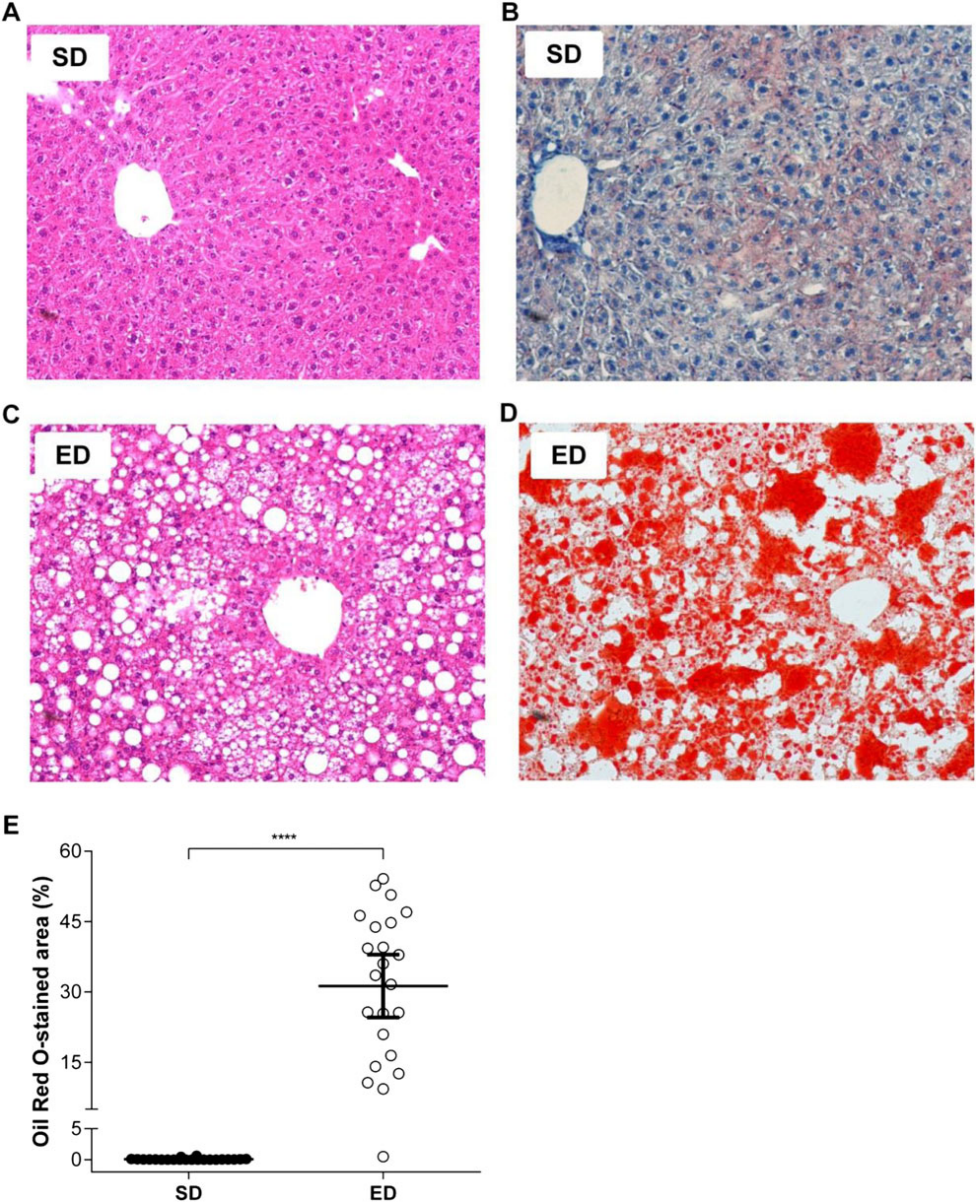
**Hepatic steatosis in mice fed an energy-dense (ED) diet.** (A-D) Photomicrographs of representative liver sections from mice fed a standard (SD; *n*=22) or ED (*n*=23) diet for 30 weeks that were stained with (A,C) H&E or (B,D) Oil Red O. 400× magnification (E) The percentage of Oil Red O staining within sections was compared using digital analysis software and demonstrates marked hepatic steatosis in ED diet-fed mice, compared with control mice. Data are mean±95% c.i. Significant differences were analysed by Student's *t*-test with Welch's correction and indicated as ***P≤0.001.

After 30 weeks of feeding, visceral and subcutaneous fat (VAT and SAT, respectively) pad masses were significantly greater for mice fed an ED diet when compared with control mice (Table 1). Furthermore, analysis of VAT morphology demonstrated an increase in the mean adipocyte size for ED diet-fed mice compared with control mice (Fig. 5A,B). In addition to marked visceral and subcutaneous adiposity, Oil Red O staining of liver revealed significant hepatic steatosis in mice fed ED diet compared with mice consuming a SD diet (Fig. 6). Our findings of dyslipidaemia, extensive ectopic fat deposits in the liver and altered fat topography are consistent with lipotoxicity described in the pathogenesis of overt T2D (Cusi, 2010; Kohlgruber and Lynch, 2015).

### Effect of ED diet on visceral adipose tissue pathology

Visceral adipose tissue (VAT) plays an important role in obesity induced insulin resistance through expression of pro-inflammatory cytokines that propagate the inflammatory response systemically and promoting insulin resistance and pancreatic β-cell exhaustion (Cusi, 2010; Kohlgruber and Lynch, 2015). Therefore, having shown significant differences in the mass of VAT between SD and ED diet-fed mice, we next compared the inflammatory cytokine profile of VAT between the two dietary groups. Compared with control mice, levels of the inflammatory cytokines MCP-1 and IL-6 were significantly elevated in VAT from ED diet-fed mice (Fig. 5C). No significant differences were observed in concentrations of TNF-α, IL-12, IFN-γ or IL-10 in VAT from mice fed a SD or ED diet for 30 weeks. Assessment of leucocyte frequencies in the stromovascular fraction demonstrated significant infiltration of immune cells into VAT from ED diet-fed mice compared with controls. Consistent with the significant expansion of VAT in mice fed an ED diet, total numbers of all leucocyte subsets assessed were increased in comparison with VAT from SD diet-fed mice. Comparison of an equivalent mass of VAT between dietary groups demonstrated significantly higher numbers of T cells and macrophages (Fig. 5E) and PMN, macrophages, NK cells, B cells, T cells, T_h_ cells and T_c_ cells (Fig. 5D-H) within VAT of ED diet-fed mice. Combined, our data are consistent with VAT inflammatory changes described for patients with T2D, together with other experimental models of T2D (Kohlgruber and Lynch, 2015; Wajchenberg and Cohen, 2014).

### ED diet feeding results in impaired renal function and pathology

**Fig. 7.**
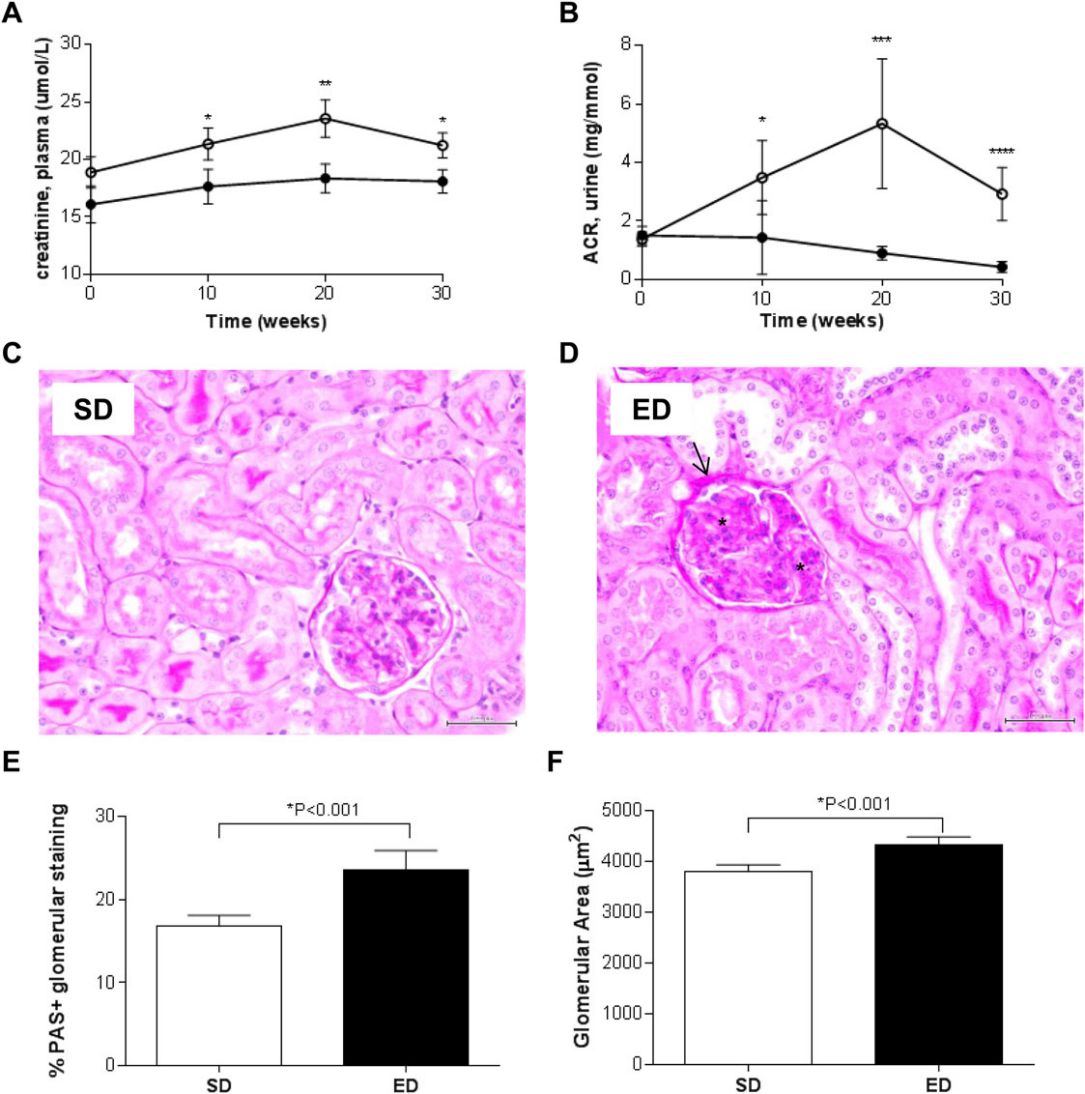
**Nephropathy in energy-dense (ED) diet-fed mice.** (A,B) Changes in (A) plasma creatinine levels and (B) urinary albumin:creatinine ratio (ACR) over 30 weeks of feeding reflect progressive impairment in renal function in mice fed an ED diet. (C,D) Representative Jones Periodic Acid Schiff's (PAS)-stained paraffin sections of kidney from mice fed a (C) standard (SD; *n*=22) or (D) ED (*n*=23) diet for 30 weeks are shown. Scale bar: 50 μm. Pronounced nephropathy was observed in kidneys from mice fed an ED diet including thickening of Bowman's capsule (arrow) and mesangial thickening (asterisk). (E) Comparison of PAS-positive staining demonstrated significant mesangial thickening within glomeruli of kidneys from ED diet-fed mice, compared with control SD-fed mice. (F) Glomerular hypertrophy was also evident in kidneys from mice fed an ED diet for 30 weeks. Data are mean±95% c.i. Significant differences were analysed by two-way ANOVA and Sidak's multiple comparison post-tests for plasma creatinine and ACR and Student's *t*-test with Welch's correction for PAS-staining. *P≤0.05, **P≤0.01, ***P≤0.001.

The impact of an ED diet on renal function was investigated through analysis of urine (glucose and albumin:creatinine (ACR) ratio) and plasma (creatinine) markers of renal impairment together with the development of renal pathology (Fig. 7, Table 1). Compared with mice fed a SD diet, those exposed to an ED diet demonstrated signs of glycosuria at 30 weeks of the intervention (Table 1). Compared with control mice, consumption of the ED diet also led to significantly elevated plasma creatinine, a marker for progressive renal failure in patients with T2D (Reddy et al., 2013) (Fig. 7A). Consistent with changes in plasma markers, the ACR was significantly higher in ED diet-fed mice at 10, 20 and 30 weeks when compared with control animals fed a SD diet (Fig. 7B). Semi-quantitative analysis of Jones Periodic Acid Schiff's (PAS)-stained glomeruli of kidneys from mice fed an SD or ED diet for 30 weeks demonstrated glomerular damage including thickening of basement membrane of Bowman's capsule (asterisk) and expansion of the mesangial matrix (arrow) (Fig. 7D,E) and hypertrophy (Fig. 7F) in ED diet-fed mice compared with control mice. A trend for a positive correlation between urinary ACR and mesangial thickening was observed in mice fed an ED diet for 30 weeks (r=0.376; *P*=0.077). These data suggest that consumption of an ED diet over a period of 30 weeks leads to development of kidney pathology and impaired renal function in C57BL/6 mice, reflective of nephropathy associated with clinical T2D.

### Effect of prolonged ED diet consumption on progression to T2D

The diagnostic criteria for clinical T2D involves a fasting plasma glucose >7.0 mmol l^−1^, a two-hour post-load plasma glucose concentration >11.1 mmol l^−1^ during a 75 g oral GTT and a HbA_1c_ >6.5% (>45 mmol mol^−1^) (American Diabetes Association, 2015; D'emden et al., 2012; The Royal Australian College of General Practitioners and Diabetes Australia, 26 Nov 2015, http://www.racgp.org.au/your-practice/guidelines/diabetes/3-screening,-risk-assessment,-case-finding-and-diagnosis/34-diagnosis-of-diabetes). In congruence with this, mice in the current study were considered T2D if they demonstrated a raised plasma glucose or HbA_1c_ with evidence of glucose intolerance at levels higher than the upper 99% confidence interval for the mean of age-matched control mice fed a SD diet. By 10 weeks, more than 90% of mice fed an ED diet demonstrated signs consistent with T2D, with similar proportions identified at 20 weeks post-dietary commencement (Table S1). Glucose intolerance was evident in 100% and elevated HbA_1c_ levels within 95.7% of mice by 30 weeks of ED diet consumption. After 30 weeks of consuming an ED diet, 82.6% of mice (19 of 23) had evidence of diabetic nephropathy based on elevated ACR and glomerular hypertrophy combined with mesangial thickening. Our findings of metabolic, inflammatory and pathological changes consistent with T2D suggest a feeding regime of 10 weeks contributes to a pre-diabetic state, whilst a regime of 30 weeks is necessary to ensure the majority of mice progress to T2D.

## DISCUSSION

The objective of this study was to evaluate whether chronic consumption of an ED diet by C57BL/6 mice leads to the development of key metabolic, immunologic and pathophysiological features akin to clinical T2D. By comparing lean mice fed standard chow with ED diet-fed mice we were able to demonstrate progressive development of metabolic and biochemical changes and the appearance of immunological and pathological modifications after a 30 week period. In addition to these pathognomonic signs of T2D, this model also reflects clinically relevant vascular complications associated with T2D. These data confirm the validity of this murine model as an accurate representation of the spectrum of pathophysiological changes associated with the development of clinical T2D.

While lifestyle factors and genetic predisposition provide an indication of those most at risk of T2D, the immunopathogenesis of this disease remains incompletely defined due to its extreme complexity, involving alterations in multiple pathways and processes. Consequently, various animal models of T2D have been reported to address knowledge gaps in the aetiology, pathophysiology, and therapeutic management of this disease. Several rodent models of T2D are described in the literature (Karasawa et al., 2009; King and Bowe, 2015; Sasase et al., 2015; Scroyen et al., 2013; Shpilberg et al., 2012; Skøvso, 2014), however, variation in the methods used to induce hyperglycaemia thwart interpretation and comparison of findings between studies. Notably, many of these models are based on high-fat diet feeding regimes which do not reflect typical global dietary patterns (Harika et al., 2013). The significant contribution of carbohydrates towards obesity, insulin resistance and T2D has become increasingly appreciated over the past decades (Dyson, 2015; Hu et al., 2000; Riccardi et al., 2008; Rizkalla, 2014; Tay et al., 2015). Consumption of ED diets, which comprise foods with a high glycaemic index, is a risk factor for development of insulin resistance and T2D (Donin et al., 2014; Gross et al., 2004). Comprehensive animal models that reflect this aetiopathogenesis are lacking. Unlike other models of diet-induced diabetes, the diet used in the current study closely reflects the typical dietary intake of developed and developing nations consisting of moderate levels of fat (20-35% of energy) in combination with increased amounts of refined sugars (>10% of energy) (Austin et al., 2011; Gross et al., 2004). The current study confirms our previous observations that compared with those consuming standard chow, male C57BL/6 mice fed an ED diet begin to develop elevated fasting blood glucose and glucose intolerance within 10 weeks of diet intervention (Hodgson et al., 2013) with the proportion of animals exhibiting diagnostic features of T2D increasing over the 30 week intervention period.

The enormous complexity surrounding the aetiopathogenesis of T2D is widely appreciated and is the focus of intense research given the emerging global impact of this chronic disease. Transition to clinical T2D is identified through a combination of fasting plasma glucose levels, an impaired oral GTT to determine insulin resistance and elevated levels of HbA_1c_ (D'emden et al., 2012). In the current study, clinical diagnostic features of T2D were apparent in the majority of mice after 10 weeks of consuming an ED diet with the proportion of animals meeting the diagnostic criteria increasing over the 30 week experimental period. Mice fed an ED diet gained significant fat mass and displayed increased fasting blood glucose, insulin resistance and elevated HbA_1c_ when compared with mice fed SD chow. At 30 weeks, HOMA-IR values revealed a ∼sevenfold increase in whole body insulin resistance for ED diet-fed mice compared with control mice, in association with increased pancreatic islet area and elevated pancreas insulin content suggestive of a compensatory hyperinsulinaemic state (Bock et al., 2003; Kahn et al., 2014).

Consistent with the pathogenic features of clinical T2D, mice fed an ED diet in the current study demonstrated a progressive increase in adiposity, circulating cholesterol and triglycerides and hepatic steatosis. Excess plasma lipids are directed to adipose tissue where increased adiposity and impairments in adipocyte function drive the establishment of an inflammatory environment with potential for systemic spillover and aggravation of the insulin-resistant state (Bays et al., 2004; Cusi, 2010; Kohlgruber and Lynch, 2015). In the clinical setting, dyslipidaemia involving high levels of plasma LDL-cholesterol and triglycerides, along with low levels of HDL-cholesterol has been associated with insulin resistance and subsequent T2D (Chehade et al., 2013). In the current study, there was evidence of higher LDL-cholesterol, HDL-cholesterol, total cholesterol and triglyceride in response to the ED diet at 10, 20 and 30 weeks following diet intervention. Whilst this appears to contrast with HDL-cholesterol profiles associated with clinical T2D, it is important to highlight that unlike humans, HDL-cholesterol is the major apolipoprotein in mice, while LDL-cholesterol only contributes to a small portion (Khovidhunkit et al., 2004). Dyslipidaemia and ectopic lipid accumulation in muscle and liver is a strong predictor of insulin resistance (Bays et al., 2004). Marked hepatic steatosis was evident in ED diet-fed mice at 30 weeks and is consistent with other rodent models of T2D (Hodgson et al., 2011; Kohli et al., 2010; Lo et al., 2011).

White adipose tissue, particularly VAT, has a fundamental role in the development of insulin resistance and T2D (Asrih and Jornayvaz, 2013), with increased adiposity associated with accumulation of macrophages and lymphocytes and increased proinflammatory cytokine secretion. In the current study, in addition to marked leucocyte infiltration, concentrations of the proinflammatory cytokines MCP-1 and IL-6 were significantly elevated in VAT from ED diet fed mice compared with control mice after 30 weeks. Local impairments in insulin signalling within VAT drives a feedforward process resulting in additional secretion of proinflammatory cytokines with systemic spill over and worsening insulin resistance in non-adipose tissue such as muscle and liver (Kahn et al., 2014; Khodabandeloo et al., 2015; Revelo et al., 2014). Similar to the clinical setting (Festa et al., 2006), plasma levels of the acute phase proteins CRP and PAI-1 and inflammatory cytokine MCP-1 were significantly higher in mice fed an ED diet compared with those fed a SD diet and is consistent with systemic low grade inflammation that is characteristic of T2D.

Many vascular complications associated with T2D arise from a combination of hyperglycaemia, AGE formation and oxidative stress, culminating in subclinical chronic inflammation and changes in the proportion of immune cell subsets and function (Cucak et al., 2014; Forbes and Cooper, 2013; Jagannathan-Bogdan et al., 2011; Olson et al., 2015). Previous clinical investigations and experimental studies have demonstrated a range of immune system impairments in patients with T2D (reviewed in Hodgson et al., 2015). Since any reduction in immune competence has to potential to lead to increased susceptibility to infection, T2D is an important risk factor for many infectious diseases, including tuberculosis (Bridson et al., 2014; Martinez and Kornfeld, 2014). There is growing interest in utilising clinically relevant animal models of T2D to investigate mechanisms of immune dysfunction that underlie susceptibility of individuals with T2D to co-morbid infectious diseases caused by pathogens such as *Mycobacterium tuberculosis* and *Burkholderia pseudomallei* (Bridson et al., 2014; Hodgson et al., 2015; Martinez and Kornfeld, 2014). In the current study, whilst the total leucocyte frequency in peripheral blood was comparable between the two dietary groups, the proportion of T_h_, T_reg_, and NKT cells was significantly lower for ED diet-fed mice than control mice which is consistent with clinical findings (Cucak et al., 2014; Jagannathan-Bogdan et al., 2011; Magalhaes et al., 2015; Olson et al., 2015; Pedicino et al., 2013). Given the importance of these cells in orchestrating protective cell-mediated immune responses that are crucial for clearance of intracellular pathogens (Hodgson et al., 2015), future investigations into the significance of this altered T cell profile in mounting subsequent effective innate and adaptive immune responses to infectious onslaughts are warranted.

Patients with T2D have an increased incidence of microangiopathies, particularly retinopathy, neuropathy and nephropathy, and are at an increased risk of cardiovascular and atherogenic diseases including atherosclerosis, myocardial infarction and cerebral ischaemic events (Fowler, 2008). The foremost complication associated with T2D is chronic renal failure which is commonly screened for in the clinical setting by measuring urine and plasma protein levels, namely microalbumin, creatinine and albumin-creatinine ratio (ACR) (American Diabetes Association, 2004; Chadban et al., 2010). The presence of microalbuminuria is highly predictive for development of end stage kidney failure and can also provide an indication of those patients most at risk of developing other vascular complications (Chadban et al., 2010). In the current study, animals fed an ED diet for 30 weeks had elevated microalbuminuria and plasma creatinine when compared with control mice fed a SD diet. Moreover, renal impairment in ED diet-fed mice was confirmed histologically with evidence of glomerular hypertrophy and glomerular basement membrane and mesangial matrix thickening, each of which are hallmark features of diabetic nephropathy (Danda et al., 2005; Jha et al., 2015; Matsubara et al., 2006). Our findings highlight the utility of this T2D model for future investigations to identify early markers of diabetic nephropathy and to assess new therapeutic targets for managing this condition.

While there were significant elevations in metabolic parameters after 10 weeks of ED diet feeding in the current study, complications of T2D require time to develop through chronic exposure to hyperglycaemia and AGE accumulation. Therefore, a 30-week feeding regime was used to not only establish metabolic dysfunction in a greater proportion of animals, but to also reflect the chronic progression of vascular complications associated with T2D. It is important to acknowledge that just as the T2D population is not a homogenous group, there was broad variation in body weight gain and metabolic changes in mice consuming an ED diet in the current study. Notably, some animals consuming the ED diet failed to develop hyperglycaemia and/or elevated HbA_1c_ within the 30-week experimental period. Our data highlights the importance of a standardised animal model of T2D with comprehensive baseline characterisation of both metabolic, biochemical and inflammatory parameters and screening of individual mice for studies utilising diet-induced models of T2D to ensure only those meeting the clinical diagnostic criteria of T2D are included for subsequent investigations. The polygenic murine model of T2D described in the current study offers several advantages to conventional T2D models: (1) use of a diet containing moderate levels of fat combined with high glycaemic index more closely reflects global dietary patterns than other models based on HFD-feeding; (2) unlike other models of T2D which use short-term diet interventions, the model described in the current study is based on chronic diet intervention and therefore not only more closely reflects the many years that it takes for progression from pre-diabetes to overt T2D and its vascular complications in humans, but also more closely simulates the mean age of diagnosis of T2D (>45 years; Age at Diagnosis, Centers for Disease Control and Prevention, 26 Nov 2015, http://www.cdc.gov/diabetes/statistics/age/fig1.htm); and (3) in contrast to previous studies describing a selective snapshot of a particular feature of T2D, the current study provides a comprehensive, longitudinal characterisation of changes in metabolic, biochemical, inflammatory, immunological and pathological markers within individual mice, thereby demonstrating the progressive development of diagnostic features of T2D.

In conclusion, data from the current study demonstrates that consumption of an ED diet consisting of moderate levels of fat and a high glycaemic index leads to the development of obesity, hyperglycaemia, insulin resistance, hyperlipidemia, chronic inflammation and impaired renal function in male C57BL/6 mice over a period of 30 weeks. This model closely simulates the progression to overt T2D, incorporating metabolic dysregulation and systemic inflammation and will provide an important tool for elucidating the molecular and cellular mechanisms involved in progression from pre-diabetes to overt T2D. In addition to its applications for studies into the pathophysiology of T2D and evaluation of glucose-lowering therapies, this model will be highly valuable for improving our understanding of the mechanisms contributing to the vascular complications associated with T2D. Given the continued global convergence of communicable and non-communicable diseases, the availability of a clinically relevant, non-genetic animal model of T2D such as that described in the current study, will also be pivotal in elucidating the immunological basis of the synergy between T2D and infections such as tuberculosis.

## MATERIALS AND METHODS

### Animals and diets

Male C57BL/6J mice were obtained from the Animal Resource Centre (Perth, WA, Australia) and housed in cages within a temperature and light controlled environment (22°C, 12 h day/night cycle). Ear tagging was used to enable longitudinal characterisation of individual mice across the study period. After two weeks of being housed with a regular diet, mice (five weeks of age) were divided randomly into two dietary groups. One group received *ad libitum* access to an ED diet [ED, *n*=23; Specialty Feeds, Glen Forrest, WA, Australia: SF03-030, 23% fat, 19% protein, 50.5% refined carbohydrates (dextrose), 7.5% fibre]. Control mice received isometric quantities of standard rodent diet (SD, *n*=22; Goldmix Stockfeeds, South Lismore, NSW, Australia: Rat and Mouse Nuts: 4.8% fat, 20% protein, 62.8% complex carbohydrates, 12.4% fibre). To control for possible differences in consumption rates due to palatability of the diets, control mice received an isometric quantity of food based on the daily consumption of paired ED diet-fed mice calculated during the previous week.

Two series of experiments were conducted in parallel. In the first series, longitudinal studies were conducted on mice fed an SD (*n*=22) or an ED (*n*=23) diet for 30 weeks. Changes in metabolic and biochemical parameters were assessed *in vivo* at baseline and at indicated time points throughout the 30 week intervention. At 30 weeks, these mice were euthanised and organs processed for histological analysis. In the second experimental series, mice were fed an SD (*n*=16) or an ED (*n*=15) diet for 30 weeks. Following confirmation of T2D status (glucose intolerance, elevated fasting blood glucose and altered plasma and urine biochemical profile), the second series of mice were euthanised, cardiac blood collected for haematological analysis and tissues processed for measurement of inflammatory cytokines and leucocyte phenotyping. Experiments were carried out in accordance with National Health and Medical Research Council guidelines and were approved by the institutional ethics committee (A2016).

### Anthropometric measurements and sample collection

The average daily food intake per mouse was calculated weekly over the 30-week dietary intervention. Body weight of mice was monitored weekly throughout the study period. At 0, 10, 20 and 30 weeks following commencement of the intervention, whole blood was collected via retro-orbital bleed from 6 h-fasted mice, with 50 μl stored for HbA_1c_ analysis and the remainder centrifuged for collection of plasma (15,000 ***g***, 10 min, room temperature). Samples were stored at −80°C until further biochemical analysis. Urine was collected from mice at baseline, 10, 20 and 30 weeks post-diet intervention and stored at −80°C until further analysis. An intraperitoneal GTT was performed on 6 h fasted mice at 0, 10, 20 and 30 weeks post commencement of the intervention.

Following completion of *in vivo* analyses at 30 weeks post-diet commencement, mice were euthanised by CO_2_ asphyxiation for end point analyses. Whole blood and plasma was collected after cardiac puncture for measurement of biochemical parameters. Liver, lung, spleen, VAT and SAT were excised, weighed and processed for histology.

### Fasting blood glucose, insulin and glucose tolerance test

Fasting blood glucose (FBG) was assessed and an intraperitoneal GTT performed on mice at baseline and at 10, 20 and 30 weeks post-dietary intervention following a 6 h fast. Blood glucose levels were measured in tail vein blood using an Accu-Chek^®^ Performa point of care blood glucose meter (Roche, Castle Hill, NSW, Australia) prior to and at 15, 30, 60 and 120 min post-injection of glucose (2 g kg^−1^ lean body mass). Hyperglycaemic threshold, as a marker for T2D, was set at 3 standard deviations above the mean (>11.0 mmol l^−1^) for control male C57BL/6 mice as described previously (Hodgson et al., 2013). The trapezoidal rule was used to determine AUC for the GTT (Hodgson et al., 2013).

At 30 weeks post-dietary intervention, plasma was separated from tail vein blood collected during the GTT and used for measurement of changes in total insulin of mice following glucose challenge. Fasting insulin levels were also measured in plasma from terminal bleed of mice at study end (30 weeks) using a commercially available Mouse Ultrasensitive ELISA kit (ALPCO, Salem, NH, USA) was used to measure total insulin in samples as per the manufacturer's instructions.

The homeostasis model assessment (HOMA) is widely used to estimate insulin resistance (HOMA-IR) (Wallace et al., 2004). HOMA-IR was calculated based on the following formulae: fasting glucose (mmol l^−1^)×fasting insulin (μU ml^−1^)/99.95. Typically, a constant of 22.5 is used for calculating HOMA-IR which is based on normal reference ranges of fasting glucose and insulin. To account for physiological differences between humans and rodents, an adjusted constant of 99.95 was used in the HOMA-IR calculation and was determined from multiplying the median levels of fasting blood glucose (11.8) and fasting insulin (8.47) from *n*=22 normal male C57BL/6 mice.

### Biochemical analyses

All biochemical analyses were performed using an AU480 Chemistry Analyser (Beckman Coulter, Mt Waverly, VIC, Australia) according to manufacturers' instructions. Total (Hb) and HbA_1c_ were measured in whole blood. Circulating glucose, triglyceride and cholesterol (total, HDL- and LDL-cholesterol), creatinine, albumin and CRP were measured in plasma. Glucose, microalbumin and creatinine concentrations were measured in urine. Total PAI-1, a marker of subclinical chronic inflammation whose expression is predictive of insulin resistance and development of T2D (Festa et al., 2006), was measured in plasma using a commercially available ELISA kit (Abcam, Melbourne, VIC, Australia) according to the manufacturer's instructions.

### Pancreatic insulin content

At study end, pancreas were excised, weighed then homogenised in 1 ml of ice-cold acid-ethanol. Homogenates were stored overnight at 4°C then centrifuged (500 ***g***, 30 min, 4°C) and the supernatant fractions transferred to fresh tubes. Samples were neutralised by addition of an equal volume of 1 M Tris pH 7.5. Insulin concentration was determined in pancreatic supernatants using a commercially available ELISA (ALPCO) and insulin content expressed as μg insulin per pancreas.

### Histology

Liver, kidney, pancreas and adipose tissue (subcutaneous and visceral) were fixed in 10% formalin for 48 h. Liver was cryosectioned (4 μm thickness) in O.C.T. medium (Tissue Tek, Olympus, Notting Hill, VIC, Australia) and stained with Oil Red O (Sigma Aldrich, Castle Hill, NSW, Australia) to evaluate neutral lipid accumulation. Kidney, pancreas and adipose tissue was embedded in paraffin and sectioned at 4 μm thickness. Sections were stained with haematoxylin and eosin (H&E, ProSciTech, Kirwan, QLD, Australia) or PAS (ProSciTech) for morphology evaluation. All stained section were visualised on a computer connected to a light microscope (BX43 Olympus). Quantitative analysis of tissues was performed on digital images (20× magnification) using cellSens™ image analysis software (Olympus). Histological analyses for each tissue were conducted on a representative section from mice that had been fed a SD (*n*=22) or ED (*n*=23) diet for 30 weeks. Threshold-based phase segmentation was used to compare the extent of neutral lipid accumulation in liver sections from each mouse. The mean size of adipocytes within VAT from each mouse was determined by measuring the area of a minimum of 50 adjacent adipocytes per section. The total pancreatic area was determined from H&E stained sections from individual mice. The total number of islets of Langerhan's per section was counted and the area of each islet measured. Data was expressed as the percentage islet area within the total pancreatic area for each mouse. In the kidney, threshold-based segmentation was applied to PAS-stained sections to compare PAS-positive staining within the glomerular capillary tuft, relative to the total glomerular area. The circumference of each glomerulus was outlined using the polygonal tracing tool and the glomerular area computed. A minimum of 30 glomeruli per kidney were assessed. Glomeruli were selected that were sectioned through the canter of the tuft and were free of artefacts. Selection bias was minimised by beginning at equivalent points in the outer cortex, moving clockwise and selecting the first acceptable glomerulus. Remaining glomeruli were selected by continuing around the cortex and selecting approximately equal numbers of glomeruli from the outer and middle zones of the cortex.

### Inflammatory cytokines

Plasma and visceral adipose tissue cytokine levels were determined by cytometric bead array [CBA; Mouse Inflammation Kit, (IL-6, IL-12p70, IL-10, TNF-α, IFN-γ and MCP-1); BD Biosciences, North Ryde, NSW, Australia]. Samples were analysed in duplicate according to the manufacturer's instructions. Data was acquired on a FACSCalibur flow cytometer using CellQuest Pro (BD Biosciences).

### Leucocyte phenotyping

Following confirmation of T2D status (elevated FBG and HbA_1c_, impaired glucose tolerance), a second series of mice fed an SD (*n*=16) or an ED (*n*=16) diet for 30 weeks were euthanised and tissues processed for leucocyte phenotyping in peripheral blood and VAT. Heparinised whole blood was also used for haematological analysis. Haematological analysis was performed on heparinised whole blood using a COULTER^®^Ac-T diff Analyser (Beckman Coulter). Using the Veterinary Applications software, the following parameters were determined: RBC and WBC count, platelet count, haematocrit (Hct), Hgb, MCH, MCHC, and MCV.

Visceral adipose tissue was excised, weighed then cut into small pieces and digested with collagenase type II (Sigma Aldrich) in Hanks Balanced Salt Solution (HBSS) with periodic mechanical disruption. The cell suspension was then filtered through 1 μm mesh and the stromovascular fraction collected after centrifugation (400 ***g***, 8 min, room temperature) and washing in HBSS containing 2.5 mM EDTA.

Cells from peripheral blood and the stromovascular fraction were pre-incubated with an anti-FcgRII/III monoclonal antibody (mAb, clone 93, eBioscience, San Diego, CA, USA) for 15 min then with appropriate mAbs. The following mAbs were used (1 in 125 dilution): fluorescein isothiocyanate (FITC)-conjugated anti-TCRβ (clone H57-597) and anti-CD3e (clone 145-2C11); Alexa Fluor (AF) 488-conjugated anti-Ly6G (clone RB6-8C5); phycoerythrin (PE)-conjugated anti-CD4 (clone RM4-5), anti-NKp46 (clone 29A1.4) and anti-MHC Class II (clone M5/114.15.2); peridinin chlorophyll (PerCP)-conjugated anti-CD45 (clone 30-F11); PerCP-Cy5.5-conjugated anti-B220 (clone RA3-6B2), anti-CD25 (clone PC61) and anti-CD11c (clone N418); allophycocyanin (APC)-conjugated anti-CD8 (clone 53-6.7); AF 647-conjugated anti-CD3 (clone 17-A2) and anti-CD11b (clone); biotinylated anti-F4/80 (clone BM8) detected with streptavidin-conjugated PE (all from eBioscience and BD Biosciences). For assessment of NKT cell frequency in peripheral blood, CD1d Dimer X was purchased from BD Biosciences and alpha galactosylceramide (α-GalCer) was purchased from Adipogen (Epalinges, Switzerland). CD1d/α-GalCer dimer was prepared according to the manufacturer's protocol and detected using PE-conjugated anti-mouse IgG_1_ (clone A85-1; BD Biosciences). Samples containing PE-labelled α-GalCer-unloaded CD1d-Ig dimer were included as controls.

Following cell surface staining, erythrocytes were lysed with RBC lysis buffer (eBioscience), cells fixed with 1% paraformaldehyde for 10 min then washed twice before resuspension in phosphate buffered saline (PBS, pH 7.4). Data was acquired on a FACSCalibur flow cytometer using CellQuest Pro (BD Biosciences). 123count eBeads (eBioscience) were added to samples immediately prior to acquisition to enable enumeration of leucocyte subsets. A minimum of 10^5^ blood leucocytes and 10^4^ leucocytes from VAT were acquired. Leucocytes were phenotyped according to the following parameters: leucocytes (CD45^+^), PMN (SSC^hi^Ly6G^+^), B cells (CD3^−^B220^+^), T cells (CD3^+^B220^−^), T_h_ cells (CD3^+^CD4^+^), T_c_ cells (CD3^+^CD8^+^), T_reg_ cells (CD3^+^CD4^+^CD25^+^), NKT cells (TCRβ^+^CD1d/α-GalCer dimer^+^), NK cells (CD3-NKp46^+^), professional phagocytic cells (CD11b^+^MHC II^+^) and tissue macrophages (F4/80^+^). The percentage of each leucocyte subset in peripheral blood from mice fed an SD or ED diet was compared, together with the absolute number of leucocytes per ml of blood. To account for the significant difference in VAT mass from SD and ED diet-fed mice, leucocyte frequencies were compared in an equivalent volume of VAT (100 mg).

### Statistical analysis

Statistical analyses were conducted using GraphPad Prism 6.0 software. Biochemical, metabolic and immunological parameters were compared between SD and ED diet-fed mice at each time point using a Student's *t*-test with Welch's correction. Data arising from repeatedly measured variables were subjected to two-way ANOVA with Tukey post-hoc test for select comparison. Correlation between glycaemic parameters (blood glucose, GTT-AUC, HbA_1c_) and other measurements were also assessed via the Spearman correlation and linear regression analysis. Data are expressed as mean±95% confidence intervals (c.i.). Significance was set at *P*≤0.05.
